# Prediction of the Impact of Land Use and Soil Type on Concentrations of Heavy Metals and Phthalates in Soil Based on Model Simulation

**DOI:** 10.3390/toxics11030269

**Published:** 2023-03-15

**Authors:** Nataša Stojić, Lato Pezo, Biljana Lončar, Mira Pucarević, Vladimir Filipović, Dunja Prokić, Ljiljana Ćurčić, Snežana Štrbac

**Affiliations:** 1 Faculty of Environmental Protection, Educons University, 21208 Sremska Kamenica, Serbia; natasa.stojic@educons.edu.rs (N.S.); mira.pucarevic@educons.edu.rs (M.P.); dunja.prokic@educons.edu.rs (D.P.); 2 Institute of General and Physical Chemistry, University of Belgrade, 11000 Belgrade, Serbia; latopezo@yahoo.co.uk; 3 Faculty of Technology Novi Sad, University of Novi Sad, 21000 Novi Sad, Serbia; cbiljana@uns.ac.rs (B.L.); vladaf@uns.ac.rs (V.F.); 4 Institute of Chemistry, Technology and Metallurgy, University of Belgrade, 11000 Belgrade, Serbia

**Keywords:** soil, impact, pollution, heavy metals, phthalates, ICP–OES, mathematical modelling

## Abstract

The main objective of this study is to determine the possibility of predicting the impact of land use and soil type on concentrations of heavy metals (HMs) and phthalates (PAEs) in soil based on an artificial neural network model (ANN). Qualitative analysis of HMs was performed with inductively coupled plasma–optical emission spectrometry (ICP/OES) and Direct Mercury Analyzer. Determination of PAEs was performed with gas chromatography (GC) coupled with a single quadrupole mass spectrometry (MS). An ANN, based on the Broyden–Fletcher–Goldfarb–Shanno (BFGS) iterative algorithm, for the prediction of HM and PAE concentrations, based on land use and soil type parameters, showed good prediction capabilities (the coefficient of determination (*r*^2^) values during the training cycle for HM concentration variables were 0.895, 0.927, 0.885, 0.813, 0.883, 0.917, 0.931, and 0.883, respectively, and for PAEs, the concentration variables were 0.950, 0.974, 0.958, 0.974, and 0.943, respectively). The results of this study indicate that HM and PAE concentrations, based on land use and soil type, can be predicted using ANN.

## 1. Introduction

A critical aspect of predicting soil pollution is the determination of the concentrations of inorganic and organic pollutants. Since examining the relationship between different soil properties and pollutant concentrations is complex, time-consuming, and expensive, in recent years, emphasis has been placed on developing models that simulate these relationships [1,2]. The main objective of this study is to determine the possibilities of predicting the impact of land use and soil type on the concentrations of heavy metals (HMs) and phthalates (PAEs) using an artificial neural network (ANN) model.

To predict the concentrations of HMs and PAEs in soil, based on the set parameters of land use and soil type, in this research, a multi-layer perceptron (MLP) model of three layers (input, hidden, and output) was applied to ANN modeling. In previous literature, the ANN model has been shown to be able to approximate nonlinear functions [3,4,5,6]. The input and output were normalized to improve the ANN behavior, while the input data was repeatedly presented to the network [7,8]. The Broiden–Fletcher–Goldfarb–Shanno (BFGS) algorithm was applied to solve the unconstrained nonlinear optimization.

To investigate the possibility of predicting the impact of land use and soil type on concentrations of HMs and PAEs, this study chose as a model agricultural soil near illegal landfills loaded with HMs and PAEs. Soil is a complex heterogeneous medium composed of mineral and organic solids and aqueous and gaseous components. Primary minerals are inherited from the parent material and are usually found in the sand and silt fraction of soils. The chemical decomposition of a rock fragment forms them. Secondary minerals are recrystallized or transformed products mainly found in the clay and fine-silt fractions. They are usually phyllosilicates or clay minerals, oxides of Fe, Al, and Mn, and sometimes carbonates (usually CaCO_3_). Soil organic matter consists of living organisms (mesofauna and microorganisms), dead plant material, and colloidal humus formed by the action of microorganisms on the plant litter [9]. Metals make up the natural constituents of the soil, but their concentrations have increased exponentially since the beginning of the Industrial Revolution [10]. The HM sources in the soil can be separated into natural and anthropogenic [11,12]. Natural sources are associated with the parent material of soil, residual organisms, and natural factors such as forest fires or volcanic eruptions [11]. Unfortunately, today, anthropogenic inputs related to natural sources are the primary sources of HMs [13]. Several HMs, such as cadmium (Cd), chromium (Cr), copper (Cu), nickel (Ni), lead (Pb), zinc (Zn), mercury (Hg), and the metalloid arsenic (As), increase their concentrations in the environment due to anthropogenic activities [14]. Mining and industrial activities produce a large quantity of Cr, Cu, and Zn [15], agricultural activities introduce Cd, Cr, and Pb into the soil [16], and traffic sources settle Cr, Ni, and Pb into the soil [17]. Domestic pollution sources lead to increased concentrations of Cd, Cr, Cu, Pb, and Zn [18]. In periurban areas, soil heavy metal pollution risks are mostly connected with the pollution effects of long-lasting mixed solid waste landfills [19]. Due to the persistence, toxicity, bioaccumulation, and biomagnification through the food chain, HMs cause environmental pollution problems [20]. Among human beings, these lead to acute toxicity, carcinogenic effects, and genotoxicity [21].

In addition to HMs, soil can be polluted by various organic compounds such as pesticides, residual crude oils, polycyclic aromatic hydrocarbons, and PAEs [22,23]. Phthalates (dimethyl phthalate, C_10_H_10_O_4_, (DMP); diethyl phthalate, C_12_H_14_O_4_, (DEP); dibutyl phthalate, C_16_H_22_O_4_, (DBP); benzyl butyl phthalate, C_19_H_20_O_4_, (BBP); and diethylhexyl phthalate, C_24_H_38_O_4_, (DEHP)) are compounds that are used in industry as plasticizers, which are added to plastics to increase their flexibility, transparency, stability, and durability. Since PAEs are not chemically linked to the polymer system, small environmental changes could accelerate the leaching, migration, or evaporation of PAEs from the plastic into the surrounding environment, harming human health [22]. Dimethyl phthalate, a known endocrine disruptor, is a colorless oily liquid with a slightly sweet odor. It is slightly soluble in water. This substance is used in cosmetics, coating products, care products, washing and cleaning products, inks and toners, laboratory chemicals, medicines, adhesives, fillers, plasters, modeling clay, varnishes, and waxes. Due to its mutagenicity, teratogenicity, and carcinogenicity, the United States Environmental Protection Agency (US EPA) has listed DMP as a priority polluter [24]. Diethyl phthalate is an odorless, colorless, oily liquid. It is used in the production of plastics, insecticides, cosmetics, toothbrushes, car parts, toys, and food packaging. As a result of its widespread use, significant human exposure to DEP is expected. Diethyl phthalate is likely to undergo environmental degradation. Degradation processes, such as hydrolysis, oxidation, and photolysis, do not significantly affect the fate of DEP in the environment [25].

Dibutyl phthalate is a colorless oily liquid insoluble in water. Due to its liquid properties, it easily penetrates the soil with the risk of groundwater pollution, representing its primary environmental impact hazard. It is mostly used as a plasticizer in resins and polymers, adhesives, printing inks, nitrocellulose paints, sealants/grouting agents, film glass fibers, and coatings. Furthermore, it has a wide range of uses in cosmetics (solvent and fixative for perfumes, lubricant for aerosol valves, suspending agent for solids in aerosols, antifoam agent, skin emollient, plasticizer in nail polish, etc.). Benzyl butyl phthalate is a clear, oily liquid. Benzyl butyl phthalate is most commonly used as a plasticizer in adhesives and sealants, floor coverings, furniture, fabrics, textiles and leather, paints and coatings, and in plastic and rubber products. Currently, BBP is banned in all toys and childcare products [26] and in cosmetics because it is considered to be carcinogenic, mutagenic, or toxic to reproduction (CMR-substance) [27]. Diethylhexyl phthalate is a colorless, oily organic carcinogen with a slight odor. It is soluble in fat and not very soluble in water. DEHP can be released into the environment during its production, distribution, processing, use, incineration, and disposal. The largest amount of DEHP in the environment is the result of the use and disposal of flexible PVC products. Inhalation, digestion, and dermal contact are the primary routes of potential exposure, which is linked to an increased incidence of hepatocellular carcinomas in animals. This substance is considered to be a human carcinogen.

They can be absorbed in all parts of the human body and even pass through the placenta, negatively affecting fetuses and newborns [28]. A strong correlation between the amount of microplastics and the concentration of PAEs has been determined [29]. Given that microplastics enter the animal and human body through various sources (cosmetics, cleaning products, clothes, polluted agriculture soil, water, etc.), it can be said that living organisms are often exposed to phthalates. The half-life of PAEs clearly indicates their persistence in the environment. The half-life of PAEs ranges from 5 h to 3.2 years [30]. Depending on pH, temperature, surfactants, pollutants, or microbial inhibitors, the half-life of PAEs in the soil can be from 1 to 75 days [31].

The main objective of this study was to determine the possibility of predicting the impact of land use and soil type on the concentrations of heavy metals (HMs) and phthalates (PAEs) in soil based on an artificial neural network model (ANN). The results of this study indicate that HM and PAE concentrations, based on land use and soil type, can be predicted using ANN. Therefore, the obtained model could be applied in the estimation of HM and PAE concentrations for the rest of Vojvodina’s unobserved sites in this study.

## 2. Materials and Methods

### 2.1. Study Area and Soil Sampling

**Figure 1:**
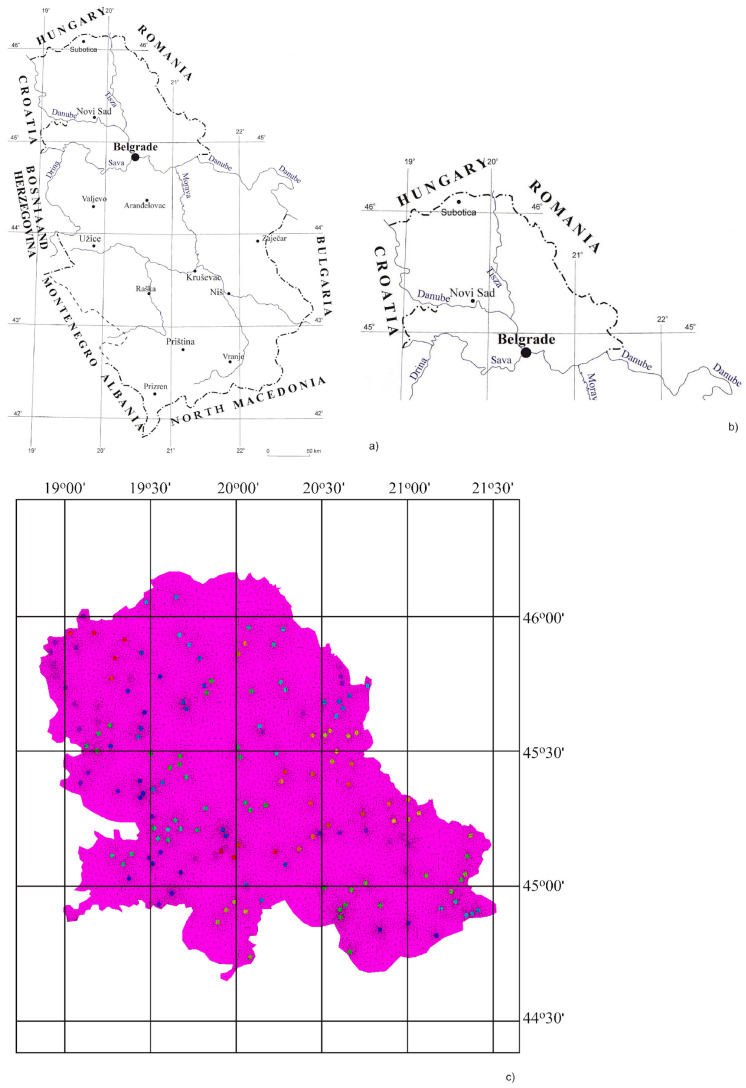
(**a**) Map of Republic of Serbia, (**b**) Map of the Autonomous Province of Vojvodina, (**c**) Distribution of HM concentrations in soil samples.

Soil samples were collected from 164 sites in the territory of the Autonomous Province of Vojvodina (APV), Serbia (Figure 1c). Vojvodina covers an area of 2,150,600 ha in the northern part of Serbia, bordering four countries (in the west with Croatia, in the east with Romania, in the north with Hungary, and in the south with Bosnia and Herzegovina) (Figure 1a,b). It is separated from the central part of Serbia by an administrative border, which consists of the Danube and Sava Rivers (Figure 1a,b). Three geographical districts, Bačka, Banat, and Srem, make up Vojvodina. Vojvodina’s geomorphology is characterized by loess, loess terraces, sand plateaus, and river plains (Danube, Tisza, and Sava Rivers). Climatic and biological factors, moderate continental climate, and steppe–forest vegetation have strongly influenced soil genesis and evolution. Overall, 1,790,000 ha or 83% of APV is agricultural land, most of which is cropland, with 1,650,000 ha or 77%. This region comprises a conventional tillage system and intensive agriculture [32].

Sampling was performed during 2017–2018. A total of 1640 soil samples were taken with a shovel at depths of 0–30 cm and 30–60 cm. The sampling tool was made of stainless steel to prevent sample contamination. Clean soil samples were packed into paper bags and transported to the laboratory. For analysis, all samples were air-dried to constant weight and homogenized to granulation ˂ 2 mm.

### 2.2. Soil Analysis

#### 2.2.1. Reagents and Standards

Acetonitrile and acetone were HPLC grade and purchased from Fisher Scientific (Fair Lawn, NJ, USA). Water was purified by an Easy pure system (Thermo Scientific, Waltham, MA, USA). Standard PAEs containing butyl benzyl phthalate, bis(2-ethylhexyl) phthalate, dibutyl phthalate, diethyl phthalate, dimethyl phthalate, and di-*n*-octyl phthalate (EPA 606-M, mix, 200 μg/mL in methanol) were purchased from Sigma Aldrich (Laramie, WY, USA). Ceramic homogenizer, dispersive SPE adsorbent (4000 mg MgSO_4_, 1000 mg NaCl, 500 mg Na_2_H citrate·1.5 H_2_O, and 1000 mg Na_3_ citrate·2 H_2_O), clean-up adsorbent (150 mg PSA, 150 mg C18, 900 mg MgSO_4_), and sodium chloride were purchased from Agilent Technologies (Lake Forest, CA, USA). For heavy metals analysis, HNO_3_ (69%, hiperpur) and H_2_O_2_ (30% *w*/*v* 100 vol.) were purchased from PanReac AppliChem (AppliChem GmbH, Darmstadt, Germany). CRM-141R—trace elements in calcareous loam soil, CRM-142R—light sandy soil (trace elements), and CRM-143R—sewage sludge amended soil (trace elements) were prepared by Joint Research Centre, Ispra, and purchased by Sigma-Aldrich Chemie GmbH (Buchs, Switzerland). Standard solutions of Cd, Cr, Co, Cu, Ni, Pb, and Zn (1000 mg/dm^3^) were purchased by J. T. Baker (SAD, Instra).

#### 2.2.2. Sample Preparation

Total HM concentrations were determined following the EPA Method 3051A [33]. Seven milliliters of concentrated HNO_3_ and 2 mL of H_2_O_2_ were added to the vessels where, previously, 0.4 g of dried and ground soil samples were briefly measured.

For the extraction of PAEs in soil samples, the QuEChERS method was used, initially developed for extracting pesticides in fruit and vegetables in 2003 [34] and extended to many other matrices and pollutants [35]. A portion of the 5 g soil sample was measured in a 50 mL centrifuge tube; 10 mL of purified water was added, followed by a ceramic homogenizer, and shaken by hand for a few seconds to hydrate the sample. After standing for 5 min, 10 mL of acetonitrile was added with 1% HCOOH and vortexed for 3 min. After that, QuEChERS mix I (extraction mix) was added and shaken for 1 min. The tube was sonicated into an ultrasonic bath for 5 min and centrifuged for 5 min at 5000 rpm. Six milliliters of supernatant were transferred into 10 mL and stored in a freezer (below −20 °C) for 2 h. The cold extract was transferred into a centrifuge tube with clean-up QuEChERS mix VI, shaken for 1 min, centrifugated for 5 min at 5000 rpm, and 2 mL of extract was evaporated to dryness under a stream of nitrogen in a 40 °C water bath. The residue was redissolved in 1 mL of acetone.

#### 2.2.3. Instrumental

For HMs analysis, soil samples were prepared using the microwave system Milestone Ethos 1 (Milestone Srl, Sorisole, Italy). Qualitative analysis was performed by inductively coupled plasma–optical emission spectrometry (ICP/OES) Varian Vista Pro-axial (Varian, Inc., Palo Alto, CA, USA). The operation conditions were a radiofrequency (RF) power of 750–1350 W, a working frequency of 1150 W, a wavelength range of 167.079 (Al)–818.326 (Na) nm, and a wavelength resolution accuracy of ±0.1 nm. The optical resolution was 189.042–327.396 nm; the atomizer flow was 0.70–0.85 L/min; the auxiliary gas flow was 0.5 L/min; the plasma gas flow rate was 0.4 L/min; and the sample pumping rate was 50 rpm. The reading delay time was 10 s, the cleaning time was 30 s, and the plasma observation method was an axial observation. Quality control was carried out with BCR reference materials CRM-141R and CRM-142R. Recoveries were within ±10% of the certified values [36]. For total Hg content, samples were analyzed using Direct Mercury Analyzer DMA 80 (Milestone). Quality control was carried out with BCR reference materials CRM-143R, and deviations were within ±5% of the certified values. Determination of PAEs was performed with gas chromatography (GC) Thermo Scientific Trace 1300, coupled with a single quadrupole mass spectrometry (MS). Operating conditions were as follows: injection volume 2 μL; MS transfer line temperature 270 °C; ion source temperature 220 °C; electron ionization mode; oven temperature 70 °C to 150 °C (25 °C/min, hold time 2 min), to 200 °C (3 °C/min), to 280 °C (8 °C/min, hold time 10 min); dwell time 0.2 min; and carrier gas helium. Quality control and quality assurance procedures were carried out using the whole procedure blank, blank spike recovery, clean soil matrix spike recovery, and comparison with reference materials. The results obtained for the recovery of PAEs ranged from 79 to 118%.

### 2.3. Data Analysis

#### 2.3.1. ANN Modeling

The experimental database for ANN was randomly divided into training and testing data (with 80% and 20% of experimental data, respectively). The training data set was used for the learning cycle of ANN and also for the evaluation of the optimal number of neurons in the hidden layer and the weight coefficient of each neuron in the network. A series of different topologies were used, in which the number of hidden neurons varied from 5 to 20, and the training process of the network was run 100,000 times with random initial values of weights and biases. The optimization process was performed based on validation error minimization. It was assumed that successful training was achieved when learning and cross-validation curves approached zero.

Coefficients associated with the hidden layer (weights and biases) were grouped in matrices *W*_1_ and *B*_1_. Similarly, coefficients associated with the output layer were grouped in matrices *W*_2_ and *B*_2_. It is possible to represent the neural network by using matrix notation (*Y* is the matrix of the output variables, *f*_1_ and *f*_2_ are transfer functions in the hidden and output layers, respectively, and *X* is the matrix of input variables [37]):

(1) $$Y=f_{1}(W_{2}\cdot f_{2}(W_{1}\cdot X+B_{1})+B_{2})$$

Weight coefficients (elements of matrices *W*_1_ and *W*_2_) were determined during the ANN learning cycle, which updated them using optimization procedures to minimize the error between the network and experimental outputs [7,38], according to the sum of squares (*SOS*) and BFGS algorithm, used to speed up and stabilize convergence [39]. The coefficients of determination were used as parameters to check the performance of the obtained ANN model.

#### 2.3.2. The Accuracy of the Model

The numerical verification of the developed model was tested using the coefficient of determination (*r*^2^), reduced chi-square (*χ*^2^), mean bias error (*MBE*), root mean square error (*RMSE*), and mean percentage error (*MPE*). These commonly used parameters can be calculated as follows [40]:

(2) $$\chi^{2}=\frac{\sum_{i=1}^{N}{\left(x_{exp,i}-x_{pre,i}\right)}^{2}}{N-n}.$$

(3) $$RMSE={\left[\frac{1}{N}\cdot \sum_{i=1}^{N}{\left(x_{pre,i}-x_{exp,i}\right)}^{2}\right]}^{1/2}.$$

(4) $$MBE=\frac{1}{N}\cdot \sum_{i=1}^{N}{\left(x_{pre,i}-x_{exp,i}\right)}^{2}.$$

(5) $$MPE=\frac{100}{N}\cdot \sum_{i=1}^{N}\left(\frac{\left\lfloor x_{pre,i}-x_{exp,i}\right\rfloor }{x_{exp,i}}\right).$$

where *x_exp_*_,*i*_ stands for the experimental values and *x_pre_*_,*i*_ are the predicted values calculated by the model, and *N* and *n* are the number of observations and constants, respectively.

## 3. Results and Discussion

### 3.1. Concentrations and Distribution of HMs and PAEs

**Figure 2:**
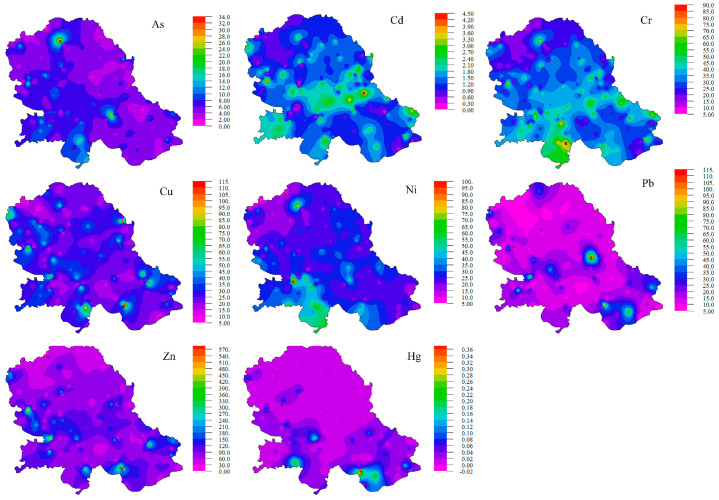
Geographical distribution of HM concentrations in soil samples.

The distribution of HM concentrations in soil samples is presented in Figure 2.

The mean ± SD (range) concentrations of As in soil samples was 6.12 ± 7.33 (0.09–140.53 mg/kg). Higher concentrations of As were observed in areas of Subotica and Zrenjanin cities (Figure 2). In most types of rocks, As is distributed rather uniformly, and its concentrations range from 0.5 to 2.5 mg/kg; only in argillaceous sediments is the average concentration high (13 mg/kg). The mean As content in soils ranges from 0.2 to 41 mg/kg [10]. At least 60% of the global atmospheric inputs of As are derived from natural sources, while human-caused sources of As are related to industrial activities.

The mean ± SD (range) concentrations for Cd and Cr in soil samples were 1.41 ± 1.0 (0.03–19.99 mg/kg) and 32.97 ± 19.73 (0.50–327.52 mg/kg), respectively. The highest concentrations of Cd and Cr were detected in augmented populated areas of Novi Sad and Zrenjanin (Figure 2). The main factor that determines the Cd content in the soil is the chemical composition of the parent rock. The highest Cd concentrations in magmatic and sedimentary rocks do not exceed 0.3 mg/kg and are mostly concentrated in shale and argillaceous deposits. The average Cd content in surface soils ranges from 0.06 to 1.10 mg/kg [10]. Higher values than average concentrations reflected the anthropogenic influence on Cd concentrations in the topsoil. In sedimentary and acidic igneous rocks, the Cr content is significantly lower and ranges from 5 to 120 mg/kg, while it is the highest in argillaceous sediments. In surface soils, the mean Cr content ranges from 7 to 221 mg/kg [10]. Due to pollution that can be caused by different sources (industrial waste, municipal waste sludge, etc.), the Cr content in the surface soil has increased.

The mean ± SD (range) concentrations for Cu and Zn in soil samples were 28.06 ± 34.35 (4.22–902.29 mg/kg) and 96.44 ± 162.89 (2.01–5418.90 mg/kg), respectively. There is a similar distribution of concentrations of Cu and Zn through the Vojvodina region (Figure 2). Parent material and soil formation processes are the two main factors that determine the initial Cu concentrations in soil. It is most abundant in intermediate and mafic rocks. In surface soils, the mean Cu content ranges from 6 to 80 mg/kg [10]. Soil Cu contamination is mainly caused by fertilizers, agricultural or municipal waste, industrial emissions, etc. Uniform concentrations of Zn occur in magmatic rocks. Slightly lower concentrations are present in acidic (40 to 60 mg/kg) rocks and slightly higher concentrations in mafic rocks (80 to 120 mg/kg). The mean Zn content in surface soils ranges from 17 to 236 mg/kg [10]. The anthropogenic enrichment of Zn is related to the nonferric metal industry, as well as to agricultural activity. The anthropogenic sources of Zn are related to the nonferric metal industry and then to agricultural practice. In certain areas, Zn pollution has led to extremely high Zn concentrations in topsoil [10].

The mean ± SD (range) concentrations of Ni in soil samples were 27.06 ± 17.30 (0.97–329.37 mg/kg). The highest concentration of Ni was obtained in the Srem region (Figure 2). The Ni contents were highest in ultramafic rocks (1400 to 2000 mg/kg). With increasing acidity in granite rocks, concentrations decrease to 5 to 15 mg/kg. Sedimentary rocks contain Ni in the ranges of 5 to 90 mg/kg. The mean Ni content of surface soils of different countries ranges from 4 to 92 mg/kg [10]. Anthropogenic sources of Ni have resulted in a significant increase in the Ni content of soils, and recently Ni has become a serious pollutant that is released in the emissions from metal processing operations and the increasing combustion of coal and oil. Important sources of Ni may also be the application of sludges and certain phosphate fertilizers.

The mean ± SD (range) concentrations for Pb and Hg in soil samples were 16.79 ± 31.11 (1.06–734.89 mg/kg) and 0.05 ± 0.12 (0.001–1.54 mg/kg), respectively. There is a similar distribution of Pb and Hg concentrations throughout the Vojvodina region (Figure 2). In the Earth’s crust, the average abundance of Pb is estimated at 15 mg/kg. The Pb tends to concentrate in the acid magmatic rocks and argillaceous sediments. The common Pb concentrations in magmatic rocks and argillaceous sediments range from 10 to 40 mg/kg, while in calcareous sediments and ultramafic rocks, their range is from 0.1 to 10 mg/kg. The mean Pb content of surface soils of different countries ranges from 7.9 to 84 mg/kg [10]. Pb is a hazardous metal to humans and animals from two sources—the food chain and soil dust inhalation; thus, the fate of anthropogenic Pb in soils has recently received much attention. A much higher concentration of Hg is reported for argillaceous sediments, sedimentary rocks, and, in particular, organic-rich shales (0.04–0.4 mg/kg). Due to widespread Hg pollution, background levels of Hg in soils are not easy to estimate. The mean Hg content in surface soil ranges from 0.02 to 0.35 mg/kg [10].

The mean concentrations of As, Cr, Cu, Ni, Pb, Zn, and Hg are below the maximum limit values prescribed by Serbian Regulation. The mean concentration of Cd exceeds the Serbian standard maximum limit values for soil (0.8 mg/kg) [41].

**Figure 3:**
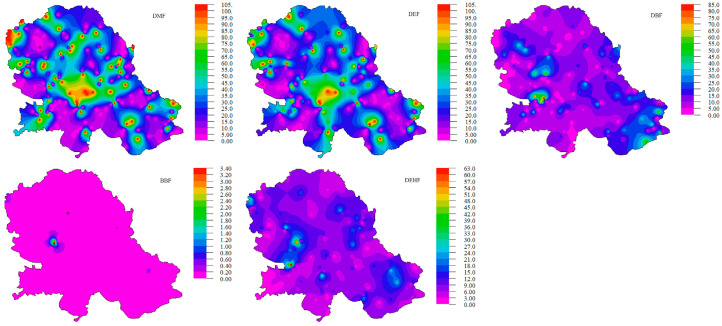
Geographical distribution of PAE concentrations in soil samples.

The distribution of PAE concentrations is presented in Figure 3.

In the presented research, the mean ± SD (range) concentration for DMP in soil samples was 2.06 ± 3.50 (0.02–43.63 mg/kg). The highest concentrations of DMP were measured in two cadastral municipalities in Vrbas and the city of Novi Sad (30.05 mg/kg and 43.63 mg/kg, respectively) (Figure 3).

Depending on the use of land, the concentration of DEP ranges from 0.0015 to 39 mg/kg. The mean ± SD (range) concentrations for DEP in soil samples in this research was 0.77 ± 1.87 (0.01–25.12 mg/kg). The highest concentrations of DEP were measured in soil samples from the municipalities of Alibunar and Plandište in South Banat District and the municipalities of Kula and Sombor in West Bačka District (Figure 3).

It exhibited relatively low acute and chronic toxicity. The mean ± SD (range) concentration for DBF in soil samples in this research was 12.26 ± 29.35 (0.01–371.43 mg/kg). Compared with the results published in the available literature, the results of these studies are about ten times higher than concentrations measured in urban areas of Port Credit (1.4 mg/kg) [42] and soil samples from vegetable greenhouses (1.118 mg/kg) [43] but much lower than the soil samples collected in the neighborhood of phthalate-emitting plants (560 mg/kg) [44].

If we exclude the cadastral municipality of Sonta, where there are very high concentrations of BBP (average value is 4053.93 mg/kg) (Figure 3), the mean ± SD (range) concentration for BBP in soil samples measured in this research was 19.18 ± 64.28 (0.02–603.77 mg/kg). The highest concentrations of BBP were measured in soil samples from the Bačka district (Bački Petrovac, Bačka Palanka, and Apatin) (Figure 3).

The mean ± SD (range) concentration for DEHP in soil samples in this research was 8.66 ± 14.92 (0.02–435.98 mg/kg), which is slightly higher than the concentration measured in soil samples from vegetable greenhouses (1.47 mg/kg) but lower than soil samples from electronics manufacturing area (21.3 mg/kg) [45]. High concentrations of DEHP in soil (129 mg/kg) were reported in the cotton fields in South Xinjiang [46]. According to the results presented in this study, higher concentrations of DEHP are observed in the higher populated cities (Figure 3).

**Figure 4:**
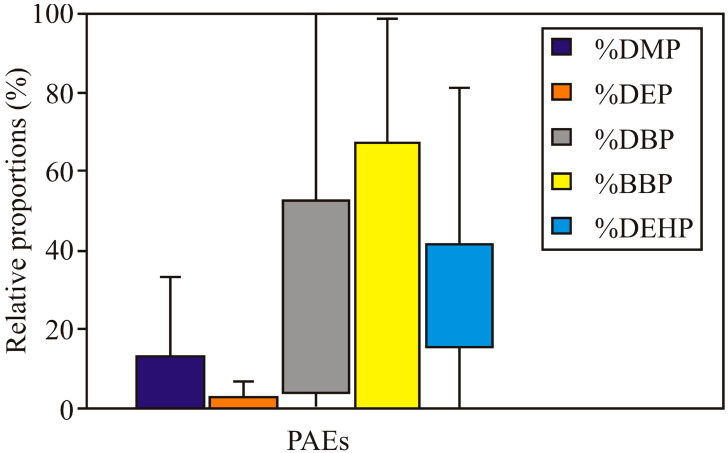
The relative proportions of the five PAEs (DMP, DEP, DBP, BBP, and DEHP) in the soils collected from 164 sites.

The concentration of total PAEs in the soil in Serbia is regulated by the Regulation on limit values of pollutants, harmful, and hazardous substances in the soil [36]. According to this Regulation, the recommended allowable soil PAE concentration is 0.1 mg/kg and 60 mg/kg for remediation value. Of all the samples, the total concentration of PAEs in soils varied from 4.59 to 667.61 mg/kg. The analysis of the results leads to the conclusion that 17% of soil samples have a concentration of total PAEs higher than the recommended remediation value (60 mg/kg), and 82.5% of samples have a concentration of PAEs higher than 0.1 mg/kg, which is the recommended maximum limit value [36]. The highest total PAE concentration was found in soil in the cadastral municipality Čelarevo in Bačka Palanka (Figure 3). PAEs may be released from a significant number of plastics and wires and come into the soil due to improper human waste disposal. The relative proportions of the five PAEs, including DMP, DEP, DBP, BBP, and DEHP, in the soils collected from 164 sites are presented in Figure 4.

The major PAEs with the largest share in the sum of the studied PAEs were BBP and DBP. This result was consistent with the reported findings [46,47] that BBP was the dominant component of the PAEs in soil. The mean ± SD (range) concentrations for the sum of five PAEs (without cadastral municipality of Sonta) was 43.07 ± 72.88 (0.026–667.61 mg/kg).

### 3.2. Prediction of the Impact of Land Use and Soil Type on the Concentrations of HMs and PAEs

The acquired optimal neural networks were used to predict the HM and PAE concentrations based on land use and soil type. Different types of land use may affect the number of used pesticides and fertilizers, which affect the concentration of HMs in the soil [48]. The capacity of different soil types to adsorb HMs is diverse [49]. Therefore, soil types can affect the migration and distribution of HMs and organic pollutants in the soil. Few published scientific papers deal with the effect of anthropogenic, climatic, and physicochemical conditions in the environment on the concentration of PAEs in contaminated soil [22]. It is considered that soil type and land use affect the transport and degradation of PAEs, and thus the vertical distribution, while precipitation and temperature are likely to affect the distribution through degradation and leaching of PAEs in the soil profile [50]. Also, a group of authors demonstrated that environmental conditions, such as temperature, soil moisture, and oxygen levels, as well as initial substance concentrations and soil type, all impact the PAE biodegradation rate [51,52]. The primary land use types in the study area included meadow, arable land, oilseed rape, soybeans, corn, fodder peas, wheat, sunflower, forest, orchard, alfalfa, vineyard, paprika, field, landfill, and the main soil types were chernozem, humogley, solonetz, solonchak, alluvium, rendzina, cambisol, and vertisol.

The acquired optimal neural networks model showed a good generalization capability for the experimental data and was used to predict the HM and PAE concentration accurately. Based on the land use and soil type, concentrations were 13 and 10, respectively, (network MLP 37-13-5 and MLP 37-10-8) to obtain the highest values of *r*^2^ (during the training cycle *r*^2^ for HM concentrations, variables were 0.895, 0.927, 0.885, 0.813, 0.883, 0.917, 0.931, and 0.883, respectively; and PAE concentration variables were 0.950, 0.974, 0.958, 0.974, and 0.943, respectively) (Table 1).

Obtained ANN models for the prediction of output variables were complex (564 and 468 weights/biases, for HM and PAE concentrations, respectively) because of the high nonlinearity of the observed system [53,54].

**Figure 5:**
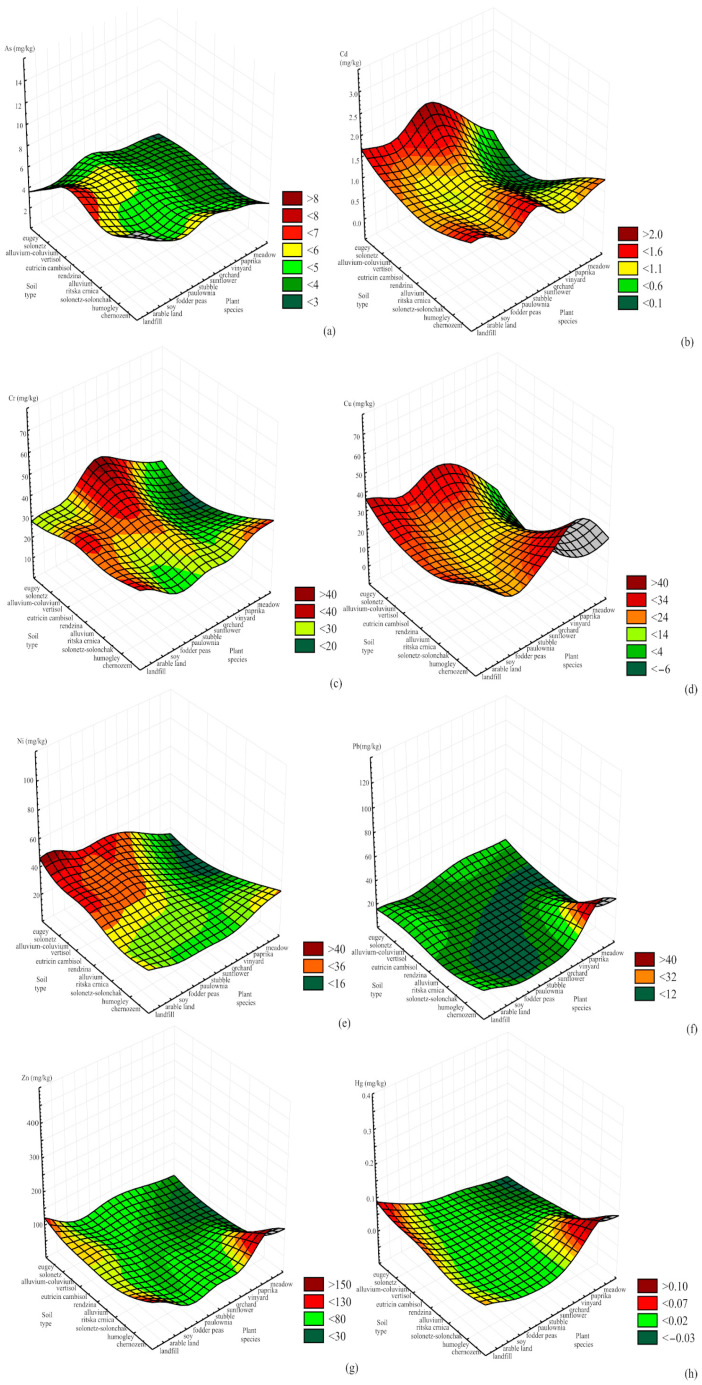
Influence of input variables on HM concentrations: (**a**) As, (**b**) Cd, (**c**) Cr, (**d**) Cu, (**e**) Ni, (**f**) Pb, (**g**) Zn and (**h**) Hg.

**Figure 6:**
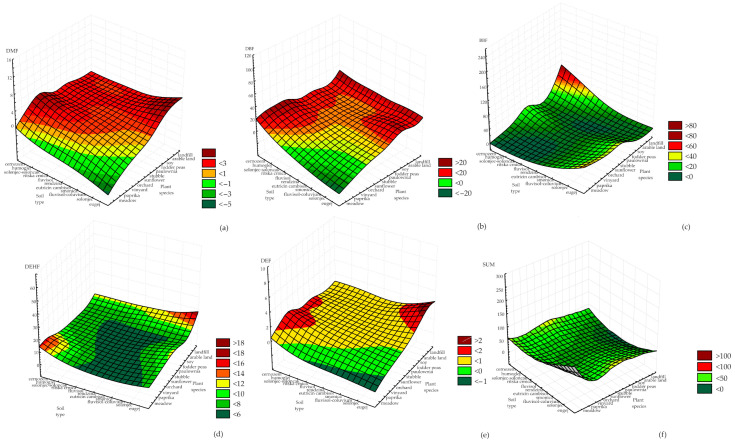
Influence of input variables on PAE concentrations: (**a**) DMF, (**b**) DBF, (**c**) BBF, (**d**) DEHF, (**e**) DEF and (**f**) SUM.

The influences of land use and soil type on HM and PAE concentrations are presented in Figure 5 and Figure 6.

The highest concentrations of Hg, Zn, and Pb were detected in samples from meadows on the chernozem soil type. Landfill samples also showed elevated Zn and Hg concentrations, but none of the samples exceeded the maximum limit value [35]. Based on previous research, the highest mean levels of Hg were reported for the histosol of Canada (0.4 mg/kg) and paddy soils of Japan (0.35 mg/kg) and Vietnam (0.3 mg/kg) [10]. Similarly, in organic and clay soils of the U.S., the highest average concentrations were found to be 0.28 mg/kg in histosol and 0.13 mg/kg in loamy soils [10]. The highest Zn mean values were reported for some alluvial soils, solonchak, and rendzina, while the lowest values were for light mineral and light organic soils [10]. Previous research has shown that only histosol is enriched in Pb, with an average value of 44 mg/kg [10]. In this study, the highest Cu, Cr, Ni, and Cd concentrations were measured on the eugej soil type. Depending on the land use, the highest concentrations of Cr and Cd were measured on stubble, of Ni on landfill, and of Cu on land used for sunflower cultivation and waste disposal. Previous research has shown that mean Cu levels range from 13 to 24 mg/kg, with the highest values in kastanozem and chernozem and the lowest in podzol and histosol [10]. The Cr content in the soil is primarily influenced by the parent rock, and the highest concentrations are present in the soil derived from mafic and volcanic rocks [10]. According to the reported data in soils on serpentines, the Cr content ranges from 0.2 to 0.4%, while sandy soils and histosol are the poorest, with an average content of 47 and 12 mg/kg, respectively. As for Ni, the highest content is in rendzina, cambisol, and kastanozem soil types i.e., clayey and loamy soils. The average content of Cd in soils lies between 0.06 and 1.1 mg/kg and seems not to correlate with the soil units, although its highest mean content is for histosol (0.78 mg/kg) and the lowest for podzol (0.37 mg/kg) [10]. The highest concentrations of As in this study were measured on the eugej soil type in soil used for soybean cultivation. The calculated lowest mean As value is 4.4 mg/kg for podzol, and the highest is 9.3 mg/kg for histosol [10].

Figure 6 shows that the concentrations of total PAEs mainly was influenced by the soil used for waste disposal, most often on the chernozem and eugej soil types.

The goodness of fit between experimental measurements and model-calculated outputs, represented as ANN performance (sum of *r*^2^ between measured and calculated output variables), during training, testing, and validation steps, are shown in Table 2.

For a wide range of process variables, the ANN model predicted the experimental variables quite well. In most cases, the predicted values were very close to the measured values in terms of *r*^2^ values. The obtained *SOS* values are of the same order of magnitude as the experimental errors for the output variables obtained in previous research [7,55]. Given that the ANN model showed an insignificant lack of fit tests, the model satisfactorily predicted the output variables. A high *r*^2^ indicates that the data fit the proposed variation model satisfactorily [56,57].

Several authors performed similar multiparameter environmental studies. Wike et al. [58] surveyed micropollutants in stormwater runoff of Berlin (Germany) and its dependence on land-use types. In a one-year monitoring program, the event means concentrations were measured for 106 parameters, including flame retardants, phthalates, pesticides/biocides, polycyclic aromatic hydrocarbons, heavy metals, and standard parameters. On the other hand, soil environmental capacity and risk warning were the core contents of soil security research in the study by Pan et al. [59], geostatistical analysis, a material balance linear model, and an environmental load capacity method were selected to simulate the ecological capacities of several heavy metals in the agricultural land of Zhongshan (China). Furthermore, numerous risks such as type, lifespan, nature of pollutants, and high cost of treatment have been associated with the treatment or remediation of contaminated soil, whether it be on-site or off-site [60].

The model obtained in this study can also be applied in similar studies regarding predictions of the impact of land use and soil type on the concentration of heavy metals and phthalates in soil.

## 4. Conclusions

The main objective of this study is to determine the possibility of predicting the impact of land use and soil type on the concentrations of HMs and PAEs in soil based on ANN. HM and PAE concentrations were analyzed in soil samples collected from 164 sites in the APV (Serbia) territory to achieve the main objective. The mean concentrations of As, Cr, Cu, Ni, Pb, Zn, and Hg are below than maximum limit values prescribed by Serbian Regulations. Only the mean concentration of Cd exceeds the Serbian standard maximum limit value for soil. The analysis of the results leads to the conclusion that 17% of soil samples have a concentration of total PAEs higher than the recommended remediation value, and 82.5% of samples have a concentration of PAEs higher than the recommended maximum limit value. Furthermore, the soils’ relative proportions of the five PAEs, i.e., DMP, DEP, DBP, BBP, and DEHP, show that BBP and DBP were the major PAEs with the largest share in the sum of studied phthalates.

The highest Hg, Zn, and Pb concentrations were detected in samples from meadows on the chernozem soil type. Landfill samples also showed elevated Zn and Hg concentrations. The highest concentrations of Cu, Cr, Ni, and Cd were measured on the eugej soil type, and depending on the land use, the highest concentrations of Cr and Cd were measured on stubble, of Ni on landfill, and of Cu on land used for sunflower cultivation and waste disposal. In this study, the highest concentrations of As were measured on the eugej soil type in soil used for soybean cultivation. The concentration of PAEs was mostly influenced by the soil used for waste disposal, most often on the chernozem and eugej soil types. 

The results of this study indicate that HM and PAE concentrations, based on land use and soil type, can be predicted using ANN, which may be helpful in future investigations on the relationship between soil parameters and pollutant concentrations.

## Figures and Tables

**Table 1:** Artificial neural network (AAN) model summary (performance and errors) for training, testing, and validation cycles.

Network Name	Performance		Error		Training Algorithm	Error Function	Hidden Activation	Output Activation
	Train.	Test.	Train.	Test.				
MLP 37-13-5	0.960	0.476	0.004	0.057	BFGS 45	*SOS*	Tanh	Exponential
MLP 37-10-8	0.892	0.642	0.023	0.054	BFGS 32	*SOS*	Exponential	Identity

Performance term represents the coefficients of determination, while error terms indicate a lack of data for the ANN model.

**Table 2:** The goodness-of-fit tests for the developed ANN model.

Output Variable	*χ* ^2^	*RMSE*	*MBE*	*MPE*	SSE	AARD	*r* ^2^
DMF	0.522	0.705	0.051	43.700	52.943	70.911	0.861
DEF	0.250	0.488	0.036	192.462	25.355	34.726	0.803
DBF	64.844	7.862	0.444	61.214	6592.970	463.722	0.757
BBF	296.758	16.819	0.059	357.363	30,268.944	1234.426	0.815
DEHF	11.585	3.323	0.526	36.427	1152.050	350.832	0.799
As	1.509	1.199	−0.043	19.718	153.734	136.480	0.747
Cd	0.067	0.253	0.000	18.963	6.876	25.846	0.791
Cr	32.772	5.589	0.458	15.202	3320.219	454.299	0.743
Cu	66.890	7.985	0.493	24.300	6796.742	641.070	0.570
Ni	43.827	6.464	0.037	17.973	4470.236	534.961	0.742
Pb	37.049	5.943	−0.420	32.281	3760.196	506.128	0.818

## Data Availability

The data will be made on reasonable request.
